# From Storybooks to Novels: A Retrospective Approach Linking Print Exposure in Childhood to Adolescence

**DOI:** 10.3389/fpsyg.2020.571033

**Published:** 2020-09-18

**Authors:** Brittany Tremblay, Monyka L. Rodrigues, Sandra Martin-Chang

**Affiliations:** Literacy Lab, Concordia University, Department of Education, Montreal, QC, Canada

**Keywords:** adolescence, print exposure, reading for pleasure, shared storybook reading, spelling, word reading

## Abstract

Despite the far-reaching advantages associated with leisure reading, it is an activity that fewer adolescents are choosing to pursue. The present study used a retrospective correlational approach to investigate shared storybook reading in childhood and current print exposure in 45 parent-adolescent dyads. Parents and adolescents completed a Retrospective Title Recognition Test, identifying storybook titles from a backdated list (books published before 2007) containing both real titles and foils. Adolescents also completed Activity Preference and Reading Enjoyment/Frequency questionnaires to assess reading habits as well as an Author Recognition Test to assess current print exposure. In addition, they were asked to name their favorite childhood storybook and favorite current author to investigate whether these two abilities were linked to print exposure. Vocabulary, reading, and spelling skills were also measured. A hierarchical multiple regression demonstrated that adolescents’ Retrospective Title Recognition Test scores accounted for unique variance in their Author Recognition Test scores, above and beyond literacy skills. Mediational analyses demonstrated that print exposure contributed to word reading and spelling scores. Our findings highlight the impact of parents’ shared storybook reading with children. Here, early reading experiences related to later reading preferences, which in turn, were associated with literacy skills in adolescence.

The accolades associated with leisure reading are impressive, including gains in spelling, vocabulary, verbal fluency, and cultural knowledge (Cunningham and Stanovich, 2001). Reading fiction specifically, correlates with increases in language skills (Mar and Rain, 2015), empathy (Nomura and Akai, 2012), and interpersonal sensitivity (Fong et al., 2013). Yet sadly, the reading habits of adolescents have been steadily declining (Twenge et al., 2019). Given the evidence supporting a reciprocal relationship between intrinsic motivation and reading volume (Schiefele et al., 2016), we took a retrospective approach to evaluate potential links between shared storybook reading from early childhood and reading habits during adolescence.

Parents have a profound influence on the home literacy environment (Sénéchal and LeFevre, 2014; Grolig et al., 2019). When reading is modeled through informal interactions with preschool children, the focus is on the enjoyable context of the storybook (Arya et al., 2014). In-line with Vygotsky’s theory (1978), children’s social interactions with knowledgeable adults can shape their later skills and behavior. Viewed from this context, the positive interactions shared during storybook reading could contribute to a propensity toward reading in later childhood (Baker et al., 1997). When reading is valued in the home, children’s enjoyment might increase, boosting intrinsic reading motivation and ultimately reading proficiency (Schiefele et al., 2012). For example, Weinberger (1996) found that 3-year-olds who could name a favorite book were better readers at age seven. However, more recent links between storybook reading and word reading have been tenuous (see Evans and Shaw, 2008 for review). Furthermore, Weinberger’s sample was not followed beyond the elementary grades thus, it remains unknown how long the influence of storybook reading extended.

Baker and colleagues (2001) found that early enjoyable shared storybook reading experiences were closely tied to children’s reading activities (as reported by parents) in Grade 3. Similarly, Sénéchal (2006) asked parents about the home literacy environment and found that children who were most exposed to storybooks in kindergarten reported reading for pleasure more often in Grade 4. Finally, as part of a 28-year study, Gottfried et al. (2015) noted that time spent reading to children (estimated by parents before age 5) had a positive direct effect on academic reading motivation and achievement in middle childhood, which in turn predicted the same factors in adolescence and educational attainment in adulthood. The authors concluded that “early reading exposure provides a foundation for subsequent long-term educational success” (p. 31). However, they did not study leisure reading during either childhood or adolescence.

Compared to self-report measures, the Title Recognition Test (TRT) offers a more objective assessment of shared storybook reading taking place in the home (Cunningham and Stanovich, 1990). The TRT was modeled after a measure of print exposure called the Author Recognition Test (ART; Stanovich and West, 1989). The ART is a proxy of reading over the lifetime. Participants are asked to identify the names of popular authors from a list containing foils. Similarly, the TRT relies on signal detection logic but it replaces author names with children’s storybook titles. Sénéchal (2000) found that the number of book titles parents recognized was positively associated with the number of characters their children recognized from book illustrations and the number of children’s books found in the home. More recently, Grolig et al. (2019) used the TRT with parents and an audiotaped TRT with preschoolers. Once again, parents’ knowledge of titles was highly predictive of children’s performance on the TRT. Both studies suggest that the TRT taps into children’s concurrent storybook reading experiences. However, neither study was designed to examine how early experiences relate to subsequent behaviors.

## The Current Study

Early social interactions may illustrate one possible difference between those who continue to read for pleasure and those who do not. To our knowledge, we are first to examine the association between shared storybook reading and print exposure into adolescence. Leisure reading has been linked to increases in academic reading motivation (Gottfried et al., 2015), reading comprehension (Torppa et al., 2020) and social competence (Kozak and Recchia, 2019), therefore it is critical to understand the factors that could be associated with it as children develop into fully literate adults. Our first aim was to examine whether shared storybook reading was correlated with print exposure in adolescence. Our second aim was to investigate whether having a favorite storybook in childhood and a favorite author in adolescence was linked to current reading habits. Finally, our third aim was to evaluate whether storybook reading, directly or indirectly via print exposure, was related to concurrent vocabulary, word reading, and spelling skills.

## Methods

### Participants

Forty-five adolescent-parent dyads were recruited via advertisements in an urban community. The parent sample consisted of 36 mothers and 9 fathers (*M*_age_ = 47.59, *SD* = 4.79). On average, parents completed 16 years of education (*SD* = 3.23) and reported English as one of their primary languages. The adolescent sample consisted of 27 females and 18 males (for descriptive statistics, see Table 1). Participants ranged from Grades 7–11 (Grade 7 *n* = 13; Grade 8 *n* = 12; Grade 9 *n* = 14; Grade 10 *n* = 1; Grade 11 *n* = 5).

**TABLE 1:** Descriptive statistics for parent and adolescent measures.

	**M (*SD*)**	**Range**	**Cronbach’s α**
Parent measures			
Storybook reading freq.	3.07(1.01)	0–4	–
R-TRT	0.27(0.20)	−0.17–0.72	0.85
Adolescent measures			
Age (years)	14.49(1.35)	12.25–17.75	–
Activity preference	1.02(1.31)	0–4	0.73
Childhood composite	20.40(11.46)	0–44	0.69
Adolescence composite	17.61(12.44)	0–48	0.83
R–TRT	0.18(0.17)	−0.30–0.52	0.82
ART	0.07(0.08)	−0.06–0.33	0.87
Vocabulary	0.22(0.18)	−0.09–0.72	0.83
Word reading^a^	105.10(14.85)	75–145	–
Spelling^b^	103.58(16.41)	70–137	–

*R-TRT, Retrospective Title Recognition Test; ART, Author Recognition Test. ^*a*^Wide Range Achievement Test-Fourth Edition. ^*b*^Woodcock Johnson-Third Edition Spelling. Three participants were missing data for the word reading task however, excluding these participants from the analyses did not change the pattern of the results.*

### Materials and Procedure

#### Parent Measures

Parents reported their birthdate, education, marital status, and language(s). They rated how frequently they read to their children before kindergarten on a 5–point Likert scale (0 = *never* to 4 = *very often*). Parents also completed the Retrospective Title Recognition Test (R-TRT) alone, without help from their child.

#### Adolescent Measures

The measures were completed in the order they appear.

##### Activity Preference Questionnaire

Participants chose between two leisure activities. Four of the nine questions involved reading (Cunningham and Stanovich, 1997). Participants received one point each time they selected reading (Max score of 4) over other activities, such as spending time on hobbies, watching television, listening to music, or playing sports.

##### Favorite Storybook/Author

Participants were asked to name their favorite storybook from childhood and their current favorite author.

##### Reading Enjoyment and Frequency Questionnaire (REF)

Adolescents reported how frequently (0 = *never* to 4 = *very often*) they engaged in leisure reading during childhood (listening to storybooks, reading chapter books, reading graphic novels) and during adolescence (reading novels, graphic novels, and non-fiction), and how much they enjoyed these activities (1 = *disliked a lot* to 4 = *liked a lot*). Childhood and Adolescence REF composites were created by multiplying frequency x enjoyment, therefore, if the frequency score was 0 for one type of reading (e.g., graphic novels), the total allotted was also 0 for that item. Each questionnaire had a maximum score of 48.

##### Retrospective Title Recognition Test (R-TRT)

Storybook reading was measured by the R-TRT (see Supplementary Appendix A). It was adapted by selecting popular children’s titles published before 2007. This ensured that all books were available by the time the youngest adolescents were born and by the time the oldest adolescents were approximately 5 years old. The backdated list was piloted with teachers for our target population. The final list contained 25 real storybook titles and 8 foils. Participants checked off each title they recognized. The proportion of checked foils was subtracted from the proportion of real storybook titles identified: (# titles correctly identified/25) – (# of foils checked/8).

##### Author Recognition Test (ART)

To assess current print exposure, adolescents completed the ART (see Supplementary Appendix B). The ART-R (Martin-Chang and Gould, 2008) was adapted to include authors of recently published adult, young adult, and children’s novels. The ART used here consisted of 110 real authors and 30 foils. Participants were alerted that guessing could be easily detected (# authors correctly identified/110) – (# of foils checked/30).

##### Vocabulary

Participants recognized words having meaning from foils. The real words with the exception of *tulip* were found in the SATs. The foils were created by combining free morphemes (e.g. *over*), bound morphemes (e.g., *ful*), roots (e.g., *rupt*), and graphemes (e.g., *eigh*) to result in non-words (see Supplementary Appendix C). The measure consisted of a list of 25 words and 18 foils. To mirror the other checklists, scores were calculated by subtracting the proportion of checked foils from the proportion of real words identified: (# words correctly identified/25) – (# of foils checked/18).

##### Word Reading

The word reading subtest of the Wide Range Achievement Test-Fourth Edition (WRAT-4; Wilkinson and Robertson, 2006) was administered. Fifty-five words were read in isolation. Testing was discontinued after ten consecutive errors. The WRAT-4 has excellent internal consistency (α = 0.92; Wilkinson and Robertson, 2006).

##### Spelling

The spelling subtest of the Woodcock Johnson Test of Achievement-Third Edition (WJ-III; Woodcock et al., 2001) was administered. Scoring was discontinued after six consecutive errors. The WJ-III has excellent internal consistency (α = 0.90; Woodcock et al., 2001).

## Results

### Preliminary Analyses

The Cronbach’s alpha for the R-TRT (parent and adolescent), ART, and vocabulary test demonstrated great internal consistency (see Table 1; Field, 2013). The Cronbach’s alpha for the Activity Preference Questionnaire and the Childhood and Adolescence REF composites were satisfactory.

On average, parents reported remembering reading to their children *often* (i.e., almost every day; see Table 1). On the R-TRT, parents rarely selected foils (*M* = 0.13, *SD* = 0.45) suggesting that they were not guessing. Their scores were also modest suggesting that they did not consult outside sources. After controlling for parents’ education, parental reports about storybook reading were positively correlated with their own R-TRT scores; parents who reported reading more often recognized more storybook titles (see Table 2). Thus, the retrospective parental measures lend support to the titles chosen for the R-TRT. Positive correlations were also noted between parents’ and adolescents’ R-TRT scores, thus providing further support for the validity of the retrospective aspect of the checklist.

**TABLE 2:** Correlations and partial-correlations between parent and adolescent measures.

	**1**	**2**	**3**	**4**	**5**	**6**	**7**	**8**	**9**	**10**	**11**
1. Storybook reading freq.	–	0.51***	−0.30*	0.07	0.26	0.40**	0.23	0.11	0.21	0.13	0.07
2. Parents’ R-TRT	0.44**	–	–0.01	0.00	0.11	0.18	0.40**	0.36**	0.15	0.00	0.14
3. Adolescent’s age	−0.34*	–0.09	–	0.03	0.02	–0.19	0.00	0.32*	0.04	–0.04	0.11
4. Activity preference	0.04	–0.07	0.02	–	0.44**	0.52***	0.41**	0.36**	0.25	0.07	0.14
5. REF Childhood	0.20	–0.03	–0.01	0.43**	–	0.71***	0.45**	0.23	0.22	0.03	–0.02
6. REF adolescence	0.36*	0.08	–0.21	0.51***	0.70***	–	0.40**	0.34*	0.08	–0.05	–0.09
7. Adolescents’ R-TRT	0.17	0.31*	–0.03	0.40**	0.41**	0.36*	–	0.48***	0.33*	0.16	0.30*
8. Adolescents’ ART	0.00	0.20	0.31*	0.35*	0.17	0.30*	0.43**	–	0.44**	0.40**	0.51***
9. Vocabulary	0.22	0.17	0.04	0.25	0.22	0.08	0.33*	0.47***	–	0.69***	0.68***
10. Word reading^a^	0.13	0.00	–0.04	0.07	0.03	–0.05	0.17	0.43**	0.69***	–	0.70***
11. Spelling^b^	0.07	0.19	0.11	0.14	–0.01	–0.09	0.31*	0.55***	0.68***	0.70***	–

Partial correlations controlling for parental education are below the diagonal. R-TRT, Retrospective Title Recognition Test; ART, Author Recognition Test. ^*a*^Wide Range Achievement Test-Fourth Edition. ^*b*^Woodcock Johnson-Third Edition Spelling. *p < 0.05. **p < 0.01. ***p < 0.001.

The Childhood and Adolescent REF composites were positively correlated with the Activity Preference Questionnaire and the amount of storybooks they were familiar with (R-TRT). However, only the Adolescent REF composite was correlated with how many authors they recognized over their lifetime (ART). These moderately strong positive correlations extend the literature by demonstrating that those who report reading more and enjoying it more, also recognize more author names.

### Linking Storybook Reading to Print Exposure in Adolescence

Our first aim was to examine whether shared storybook reading during childhood (R-TRT) would be correlated with print exposure in adolescence (ART and Activity Preference Questionnaire). As shown in Table 2, after controlling for parental education, both measures were positively correlated with the R-TRT. Therefore, when adolescents recognized more storybook titles, they were more likely to choose leisure reading over other activities and recognize more authors.

A hierarchical multiple regression was conducted to examine the association between the R-TRT and the ART (see Table 3). To address the multicollinearity between the literacy measures and reduce the number of predictors entered in the regression, we created a composite by averaging the z-scores of the three literacy measures. After parental education, adolescents’ age, and literacy skills accounted for 40% of the variability in adolescents’ ART scores, the R-TRT scores still explained 8% of unique variance, suggesting that shared storybook reading during childhood may play a role in shaping print exposure into adolescence.

**TABLE 3:** Hierarchical multiple regression analysis estimating associations with the ART.

	***R^2^_c_***	***F***	***B***	***SE***	**β**	***t***
ART	0.40	10.25***				
Parental education			0.01	0.01	0.32	2.67**
Adolescent’s age			0.02	0.00	0.27	2.25**
Literacy composite^a^			0.05	0.01	0.50	4.12***
	0.48	6.17**				
R-TRT			0.14	0.06	0.31	2.48**

R-TRT, Retrospective Title Recognition Test; ART, Author Recognition Test. ^*a*^The three literacy measures were z-standardized, summed and divided by three. **p < 0.01. ***p < 0.001.

### Favorite Storybook/Author

Our second aim was to determine if there were differences in reading habits between those who named a favorite storybook or author and those who did not. Approximately 49% of adolescents named a favorite storybook title and 40% named a current favorite author. There was a difference in all means between participants who named a favorite author versus those who did not. Identifying a favorite author was linked to: (1) choosing reading over other activities, *U*(43) = 140.00, *z* = −2.58, *p* = 0.01, *r* = −0.38 (*M*_1_ = 1.61, *SD*_1_ = 1.46; *M*_2_ = 0.63, *SD*_2_ = 1.04^1^; (2) reporting greater reading enjoyment and frequency during childhood, *t*(43) = −3.39, *p* < 0.001, *g* = 1.13 (*M*_1_ = 26.78, *SD*_1_ = 11.35; *M*_2_ = 16.15, *SD*_2_ = 9.55) and adolescence, *t*(42) = −3.21, *p* = 0.01, *g* = 0.99 (*M*_1_ = 24.17, *SD*_1_ = 11.85; *M*_2_ = 13.08, *SD*_2_ = 10.80), (3) recognizing more storybook titles on the R-TRT, *t*(43) = −2.82, *p* = 0.01, *g* = 0.88 (*M*_1_ = 0.26, *SD*_1_ = 0.05; *M*_2_ = 0.12, *SD*_2_ = 0.17), and (5) recognizing more authors on the ART, *t*(43) = −3.62, *p* < 0.001, *g* = 1.15 (*M*_1_ = 0.12, *SD*_1_ = 0.09; *M*_2_ = 0.04, *SD*_2_ = 0.05). Overall, these findings support the sensitivity of the single-item measure in separating adolescents who read frequently and infrequently. Similar analyses with favorite storybook yielded null results.

### Print Exposure and Literacy Skills

Finally, we investigated whether storybook reading and print exposure were linked to concurrent literacy skills. Adolescents’ shared storybook reading (R-TRT) was positively correlated with their spelling and vocabulary, but not with their word reading (see Table 2). Their print exposure (ART) scores were moderately positively correlated with all three literacy measures. Thus, we investigated the associations between the literacy skill outcomes and print exposure scores on the R-TRT and ART in a set of mediational analyses. Of note, each of the literacy skills showed slight multicollinearity with one another (Field, 2013). Thus, the analyses did not control for each literacy skill, permitting a more direct evaluation of the associations.

#### Mediation Analyses

**FIGURE 1:**
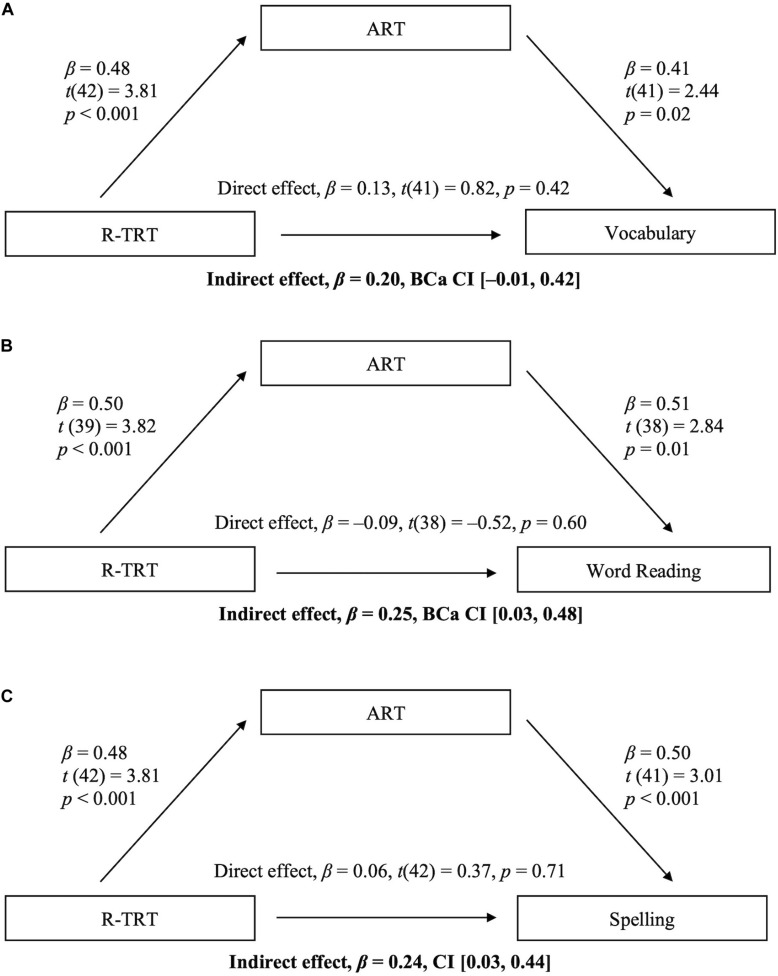
Mediational model for the association between the R-TRT and literacy skills mediated by the ART (controlling for adolescent’s age).

To test our hypothesis that early storybook reading (R-TRT) is related to later literacy skills through the support of current print exposure (ART), we submitted the scores for the R-TRT, ART, and the three outcome variables to three separate mediational analyses while also controlling for age (see Figure 1). The analyses were carried out in IBM SPSS Statistics v26 using the *PROCESS* macro v3.2 (Hayes, 2018). The bootstrap procedure, which is well suited for small-scale studies, computed confidence intervals for mediated effects based on 5,000 resamples (Preacher and Hayes, 2004; Field, 2013).

The indirect association between the R-TRT and vocabulary (through its effect on current print exposure) failed to reach significance. As seen in Panel A, the bootstrapped 95% bias-corrected confidence interval for the completely standardized indirect effect just touched zero. In contrast, as seen in Panels B (word reading) and C (spelling) the R-TRT was indirectly associated with both word reading and spelling through its effect on current print exposure. This indicates that participants who were exposed to more storybooks as children were more likely to read for pleasure as adolescents and in turn, participants who were more likely to read for pleasure were better at word reading and spelling. The bootstrapped 95% bias-corrected confidence interval for the completely standardized indirect effects were above zero and were supported by small effects of *R*^2^*_m__ed_* = 0.09 and *R*^2^*_m__ed_* = 0.08, respectively (Fairchild and McQuillin, 2010; Hayes, 2018). This indicated that storybook reading did not affect word reading or spelling independent of its effect on print exposure.

## Discussion

The overarching goal of our study was to investigate whether shared storybook reading in childhood was associated with print exposure in adolescence. Although researchers often allude to the importance of shared storybook reading on children’s emerging language skills and its impact on child enjoyment (e.g., Sénéchal and LeFevre, 2014; Patel et al., 2020), to our knowledge no studies have examined these relationships into adolescence or beyond. We also explored whether remembering a favorite storybook from childhood or having a favorite author as a teenager mirrored having a favorite storybook as a child (Weinberger, 1996). Finally, we aimed to replicate the links between print exposure and concurrent literacy skills in a sample of adolescents.

Baker and colleagues (1997) contend that early pleasurable shared storybook experiences are at the root of children’s feelings about reading and their eventual desire to read. The present study provides the first empirical evidence that we know of, linking parental reports of storybook reading and adolescents’ self-reported reading enjoyment and frequency. Using a more objective measure, we also noted that adolescents who recognized more storybook titles from childhood, were also able to recognize more authors of children’s, young adult, and adult novels. Furthermore, Weinberger (1996) first suggested that these early enjoyable experiences contributed to reading ability and to children’s involvement with reading for pleasure as they age. In our data, the more storybook titles adolescents recognized from childhood, the more they chose reading over other activities in high school. This pattern extended to current reading habits; adolescents who recognized more authors also reported holding more favorable views toward reading as measured by the Adolescent REF composite.

Electing to read for pleasure is a personal choice that may be related to many factors, however, based on our results, early shared storybook reading could be one of them. Even after employing very stringent controls, shared storybook reading accounted for unique variance in leisure reading. These findings are uniquely compelling because of the retrospective nature of the R-TRT. The fact that identifying titles published 13 years prior (at minimum) was able to predict present day ART scores suggests that storybook reading not only promotes language development in childhood (Nyhout and O’Neill, 2013), but may pave the way to reading for pleasure into adolescence.

Another result to acknowledge stems from adolescents’ ability to name their favorite storybook from childhood and their current favorite author. In the present study, recalling a favorite storybook without prompting (as opposed to recognizing titles in the R-TRT) did not differentiate the two sub-samples on any of the variables of interest. This was most likely due to the amount of time that had passed since childhood. Being asked about a participant’s current favorite author, however, did broadly separate adolescents who read more from those who read less. Those who named a favorite author chose reading over other activities, reported higher enjoyment and frequency of reading in childhood and adolescence (REF scores), and scored higher on both proxies of print exposure (R-TRT and ART). This places our study among the ranks of others that have noted the efficacy of single items in predicting behavioral outcomes (e.g., Gardner et al., 1998; Hoeppner et al., 2011). Thus, naming a favorite author may be a useful initial assessment for teachers, as this single item was linked to students’ prevalent reading habits.

Our findings also align with previous research on the association between shared storybook reading and educational standings (e.g., Gottfried et al., 2015); in our study participants who recognized more storybook titles outperformed their peers on vocabulary and spelling measures. Word reading, on the other hand was not correlated with performance on the R-TRT. This finding was foreshadowed by the literature showing that shared storybook reading is either less positively correlated with emerging reading skills or in some cases negatively correlated with them (e.g., Evans and Shaw, 2008). In contrast, print exposure, which involves individuals actively reading themselves, shows robust associations with reading and spelling skills (Martin-Chang et al., 2020). Therefore, we were not surprised that the R-TRT showed no direct associations to current literacy skills in the mediational models. As expected, the R-TRT was indirectly associated to word reading and spelling through the ART and, although not significant, the same trend was noted for vocabulary. Taken together, a broad pattern emerges where participants who were exposed to more storybooks as children, showed a greater inclination to read for pleasure and in turn, had more advanced literacy skills as adolescents. These effects were modest, explaining between 8 and 9% of the skills in question, yet we would argue they are nonetheless meaningful. The literacy skills under consideration are complex and have been associated with both genetic predispositions (Friend et al., 2007) and other environmental factors such as quality of schooling (Petrill et al., 2010). Thus, we feel the results discussed here are worth highlighting because increasing storybook reading is easily amenable to change.

Our findings carry two implications. First, they suggest that children who experience reading with a caregiver are more likely to read independently once their reading skills develop. Second, while speculative, it proposes that children who missed the opportunity for shared storybook reading may make up for lost time by choosing to engage in independent reading as they grow. Thus, influential adults (e.g., teachers, extended family, and tutors) should continue to promote reading as an entertaining and worthwhile activity. Likewise, it recommends that parents’ jobs as reading partners do not end when their children become too big to sit on their laps. Rather, parents should scaffold reading using storybooks when their children are young and encourage the progression toward reading chapter books and novels independently as their children’s skills develop.

The innovative design of our research offers two new contributions to the literature. We demonstrated a relation between shared storybook reading in childhood and an inclination toward reading into adolescence. By extension, it is also the first to compare the relative influence of shared storybook reading versus leisure reading with regards to literacy skills. Further, we created a retrospective measure that avoided many of the complications associated with longitudinal designs, such as the time and cost of tracking participants throughout their lives. The retrospective nature of the task should be interpreted with caution and used alongside corroborating measures. However, because parents’ R-TRT scores were correlated with their children’s (albeit slightly weaker than concurrent TRT scores within parent child dyads; cf. Grolig et al., 2019), it supports the validity of measuring shared storybook reading retrospectively. The R-TRT offers researchers a glimpse into participants’ home literacy environments that was previously unavailable.

### Limitations

A potential limitation of this study is the small sample size. Small sample sizes reduce statistical power and make finding effects more difficult. Even under the current conditions the analyses yielded reliable results. Nonetheless, due to the risk of Type 1 error, future work should aim to replicate these patterns with larger samples. A second issue is the absence of parents’ socioeconomic status (SES). Manolitsis et al. (2013) studied the home literacy environment and found that SES had no effect on formal and informal literacy activities. Similarly, the kinds of literacy activities parents use with their children generalize across SES (Hood et al., 2008). We did, however, include parent’s education, which is a reliable predictor of both reading materials found in the home and time spent reading with children (Gottfried et al., 2015). A third limitation is the low recognition rate on the ART. Future studies should consider using the ART-CYA which was created for children and young adults (Martin-Chang et al., 2020).

Another limitation to consider is the correlational nature of the study. Although there was a positive correlation between the number of storybook titles adolescents identified and the number of authors recognized, it could be that the association was mediated by an unmeasured variable. Perhaps adolescents who read to younger children or who place importance on reading, also value their memories of shared storybook reading and therefore are able to identify more storybook titles (e.g., perhaps they still own their storybooks as keepsakes). In addition, it could be that parents who read more storybooks to their children in the early years continued to promote reading as their children grew. Future research should ask adolescents about the role parents, teachers, and peers play in supporting leisure reading, and whether structural support, such as easier access to books via libraries (onsite or online) could promote reading.

### Implications

When parents and children share storybooks, the goal often includes engaging in meaningful experiences (Arya et al., 2014). Vygotsky’s Sociocultural Theory (1978) asserts that children develop behaviors and learn social norms through their interactions with more competent individuals. Parents are scaffolding book reading during these social interactions as they model concepts about print (Sénéchal and LeFevre, 2002) as well as higher order thinking through discussion and enjoyment (Patel et al., 2020).

Shared storybook reading is associated with many concurrent benefits, including heightened vocabulary (Flack et al., 2018) and advanced theory of mind (Mar, 2018). Our findings suggest that shared storybook reading may also support children’s subsequent print exposure and reading preferences into adolescence. Therefore, parents should be encouraged to luxuriate in shared storybook reading as it may very well instill a long-lasting love of reading into adolescence and beyond.

## Data Availability Statement

The raw data supporting the conclusions of this article will be made available by the authors, without undue reservation, to any qualified researcher.

## Ethics Statement

The studies involving human participants were reviewed and approved by the Office of Research, Concordia University. Written informed consent to participate in this study was provided by the participants’ legal guardian/next of kin.

## Author Contributions

BT spearheaded this research project, designed the R-TRT, and collected and analyzed the data for her master’s thesis, all under the supervision of SM-C. MR collaborated on the data analysis. All authors adapted BT’s thesis for this article and approved the final version of the article for publication.

## Conflict of Interest

The authors declare that the research was conducted in the absence of any commercial or financial relationships that could be construed as a potential conflict of interest.

## Footnotes

1 The data from the Activity Preference Questionnaire were positively skewed, justifying nonparametric tests (Field, 2013).
