# Supplementary material for: From Storybooks to Novels: A Retrospective Approach Linking Print Exposure in Childhood to Adolescence
Source: Front Psychol. 2020 Sep 18;11:571033. doi: 10.3389/fpsyg.2020.571033 (PMC7531176; doi:10.3389/fpsyg.2020.571033)
Supplement: Supplementary file 1 [file Data_Sheet_1.pdf]

Appendix A

Retrospective Title Recognition Test (R-TRT)

Children's Book Titles	Adolescent Hit Rate (%)	Parent Hit Rate (%)
Flat Stanley	60.0	55.6
Where the Wild Things Are	55.6	48.9
The Hockey Sweater	53.3	46.7
Are You My Mother?	48.9	42.2
Goodnight Moon	48.9	62.2
Because I Love You	46.7	28.9
Chicka Chicka Boom Boom	46.7	57.8
Oh, the Places You'll Go!	46.7	46.7
Brown Bear, Brown Bear, What Do You See?	40.0	46.7
If You Give a Pig a Pancake	37.8	13.8
Harold and the Purple Crayon	31.1	13.3
Biscuit	24.4	20.0
Click, Clack, Moo: Cows That Type	24.4	15.6
Danny and the Dinosaur	24.4	13.3
Dog Heaven	24.4	6.7
Guess How Much I Love You	24.4	51.1
Father Bear Comes Home	22.2	4.4
Bartholomew and the Oobleck	20.0	8.9
The Story of Ferdinand	20.0	11.1
The Going to Bed Book	13.3	6.7
Grandma and the Pirates	11.1	17.8
The Runaway Bunny	8.9	15.6
Caps for Sale	6.7	2.2
Corduroy	6.7	35.6
Gerald McBoing Boing	4.4	2.2

Foil Items	Adolescent False Alarm Rate (%)	Parent False Alarm Rate (%)
Lazy Cat, Lazy Cat	22.2	4.4
The Muffin Maker	20.0	0.0
Blame it on Billy	15.6	0.0
Clean up, Carter!	11.1	0.0
My Friend the Mailman	8.9	2.2
What Rhymes with Orange?	8.9	4.4
Down by David's Pond	6.7	0.0
Wacky Wendell	4.4	0.0

Appendix B

Author Recognition Test (ART)

Authors	Hit Rate (%)	Authors	Hit Rate (%)
J.K. Rowling	91.1	Mary Higgins Clark	4.4
Stephen King	73.3	Anthony Horowitz	4.4
Judy Blume	51.1	Marc Levy	4.4
John Green	44.4	Marissa Meyer	4.4
Suzanne Collins	42.2	Alice Munro	4.4
Roald Dahl	37.8	Dav Pilkey	4.4
C.S. Lewis	26.7	Louis Sachar	4.4
James Patterson	26.7	Robert J. Sawyer	4.4
J.R.R. Tolkien	26.7	Patrick Senécal	4.4
Mélanie Watt	26.7	Ginette Anfousse	2.2
Agatha Christie	24.4	Russell Banks	2.2
Tom Clancy	24.4	Patricia Cornwell	2.2
Rick Riordan	24.4	Gayle Forman	2.2
Jeff Kinney	22.2	John Grisham	2.2
James Dashner	17.8	Shannon Hale	2.2
RJ Palacio	17.8	Frank Herbert	2.2
Dan Brown	15.6	Erin Hunter	2.2
Meg Cabot	15.6	Robert Jordan	2.2
Stephenie Meyer	15.6	Sophie Kinsella	2.2
L.M. Montgomery	15.6	Pittacus Lore	2.2
Jerry Spinelli	15.6	Marie Lu	2.2
Margaret Atwood	13.3	Sarah J. Maas	2.2
Cassandra Clare	13.3	Ann Marie MacDonald	2.2
Alexandre Dumas	13.3	Guillaume Musso	2.2
Gordon Korman	13.3	Katherine Paterson	2.2
Jay Asher	11.1	Nora Roberts	2.2
Cornelia Funke	11.1	Sonia Sarfati	2.2
François Gravel	11.1	Sara Shepard	2.2
George R.R. Martin	11.1	Maggie Stiefvater	2.2
Michael Moore	11.1	Miriam Toews	2.2
David Baldacci	8.9	Leigh Bardugo	0.0
John Flanagan	8.9	Robin Benway	0.0
Laurie Halse Anderson	8.9	John Boyne	0.0
Lois Lowry	8.9	Ann Brashares	0.0
Ann M. Martin	8.9	Sharon Creech	0.0
Christopher Moore	8.9	Hélène Desputeaux	0.0
Gary Paulsen	8.9	Kate DiCamillo	0.0
Marie Hélène Poitras	8.9	Jilly Gagnon	0.0
Rainbow Rowell	8.9	Claudia Gray	0.0
Danielle Steel	8.9	Laurell K. Hamilton	0.0
Elizabeth George	6.7	John Jakes	0.0

Authors	Hit Rate (%)	Authors	Hit Rate (%)
E.L. James	6.7	Dean Koontz	0.0
Kenneth Oppel	6.7	Margaret Laurence	0.0
Kathy Reichs	6.7	Louise Leblanc	0.0
Lemony Snicket	6.7	Ursuala LeGuin	0.0
Raina Telgemeier	6.7	Lauren Oliver	0.0
Gilles Tibo	6.7	Christopher Paolini	0.0
V.C. Andrews	4.4	Jacques Poulin	0.0
Katherine Applegate	4.4	Philip Pullman	0.0
Isaac Asimov	4.4	Anne Rice	0.0
Eoin Colfer	4.4	Mordecai Richler	0.0
Jackie Collins	4.4	Rachel R. Russell	0.0
Diana Gabaldon	4.4	Amy Tan	0.0
Sue Grafton	4.4	Laini Taylor	0.0
Élise Gravel	4.4	AlvinToffler	0.0

Foil Item	False Alarm Rate (%)
Katherine Carpenter	6.7
Chloe Lamoureux	6.7
Phillippe Brisebois	4.4
Suzanne Clarkson	4.4
W. Patrick Dickson	4.4
Marc Geofferion	4.4
Sheryl Green	4.4
Mélanie Lachance	4.4
James Morgan	4.4
Christopher Barr	2.2
Elliot Blass	2.2
Edward Cornell	2.2
Robert Emery	2.2
Jean Lajoie	2.2
Alain Levesque	2.2
Danielle Page	2.2
Allan St. Pierre	2.2
Mark Strauss	2.2
Lauren Benjamin	0.0
Thomas Bever	0.0
Jennifer Butterworth	0.0
Frank Kiel	0.0
Luc Larouche	0.0
Priscilla Levy	0.0
Alex Lumsden	0.0
Morton Mendelson	0.0
David Perry	0.0
Miriam Sexton	0.0
Destin Shaw	0.0

Robert Siegler	0.0

Appendix C

Vocabulary Test

Word	Hit Rate (%)	Foil Item	False Alarm Rate (%)
tulip	86.7	strickling	28.9
antic	66.2	burntout	26.7
elliptical	53.3	carace	26.7
molten	51.9	gradful	24.4
optician	51.1	ruptious	24.4
shun	51.1	overplea	22.2
abode	48.9	sall	17.8
bask	44.4	dreep	15.6
coax	44.4	farlist	11.1
bugle	40.0	eighful	8.9
ramification	40.0	mooror	8.9
tensile	40.0	aphe	6.7
canter	37.8	chalipmentary	6.7
sundry	35.6	rew	6.7
dinghy	33.3	thouple	6.7
fallacy	28.9	avire	4.4
copious	26.7	klight	4.4
postulate	26.7	toulir	2.2
guise	24.4		
poignant	24.4		
waggish	20.0		
dirge	13.3		
impetus	11.1		
aver	4.4		
verve	4.4		
